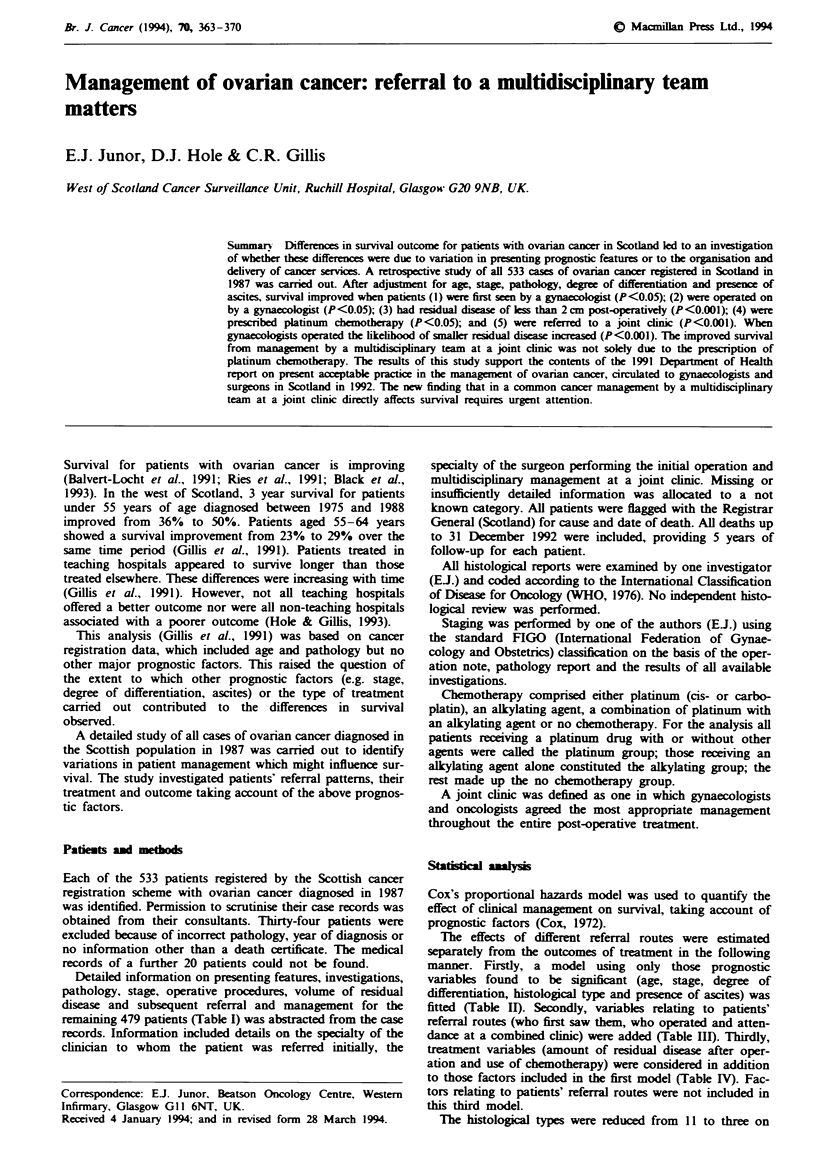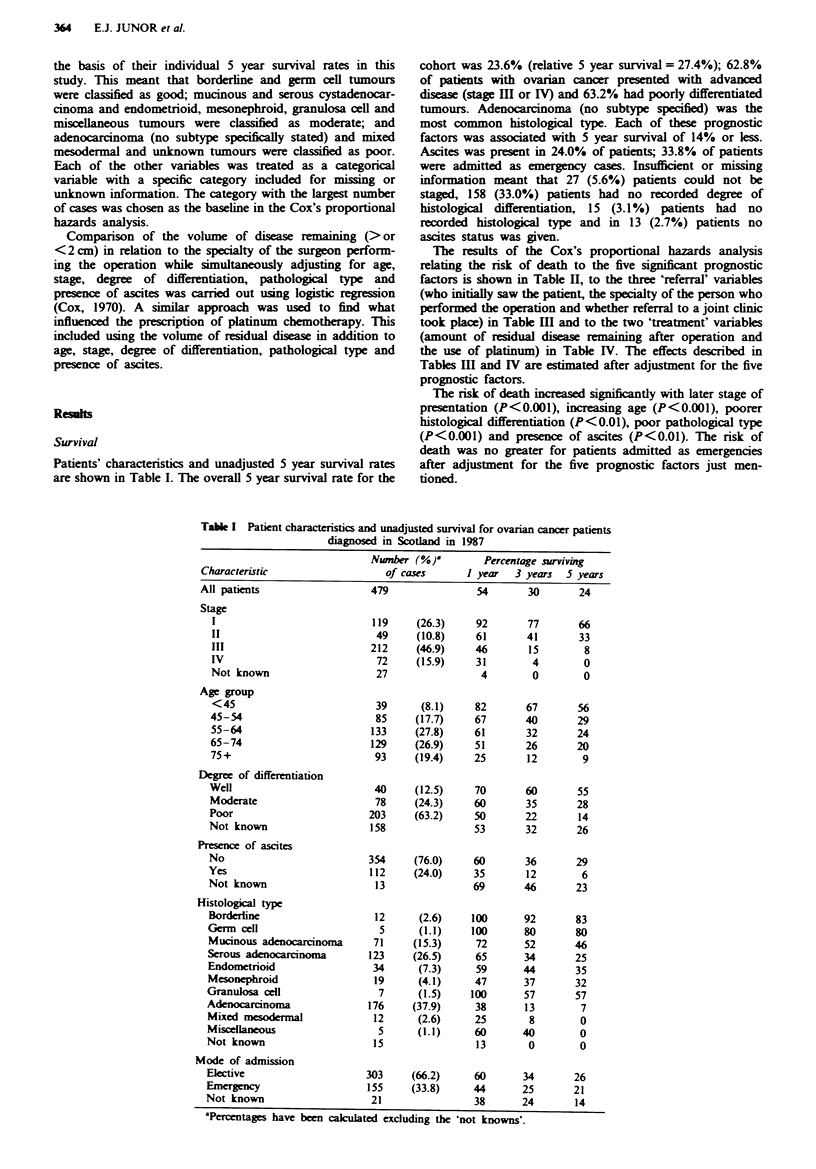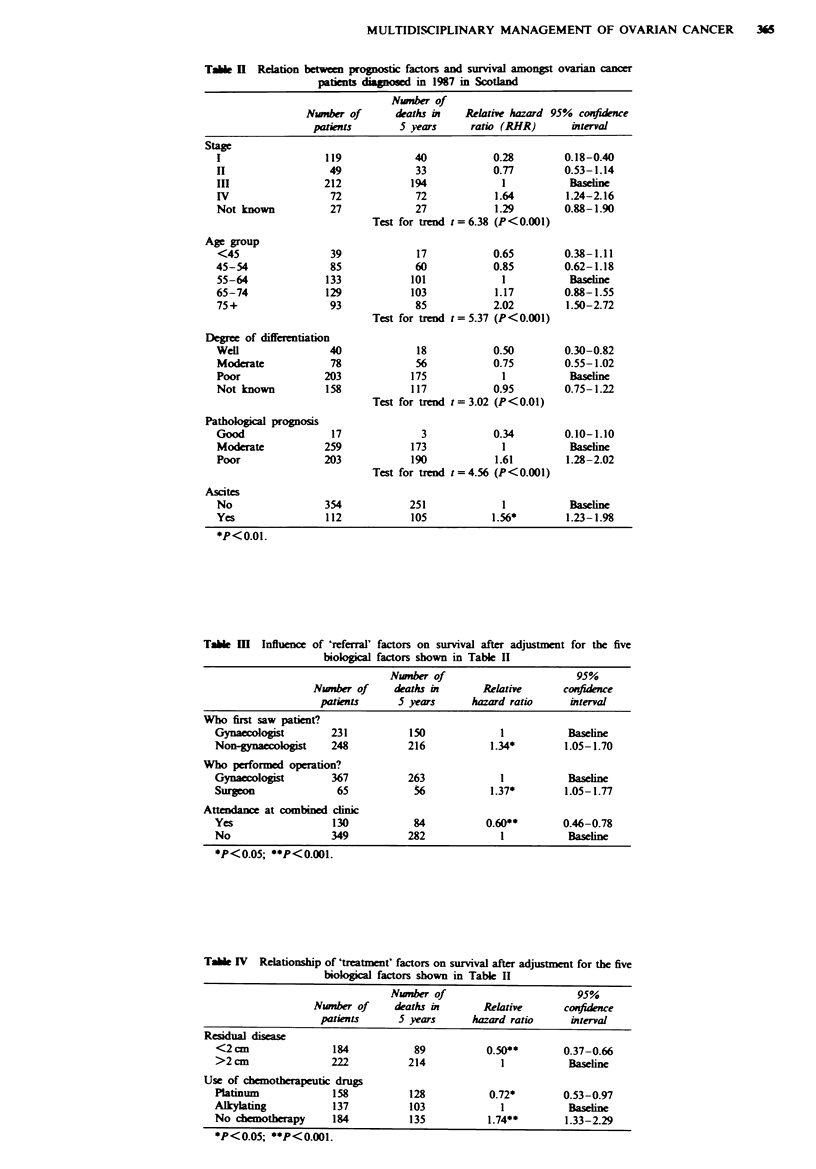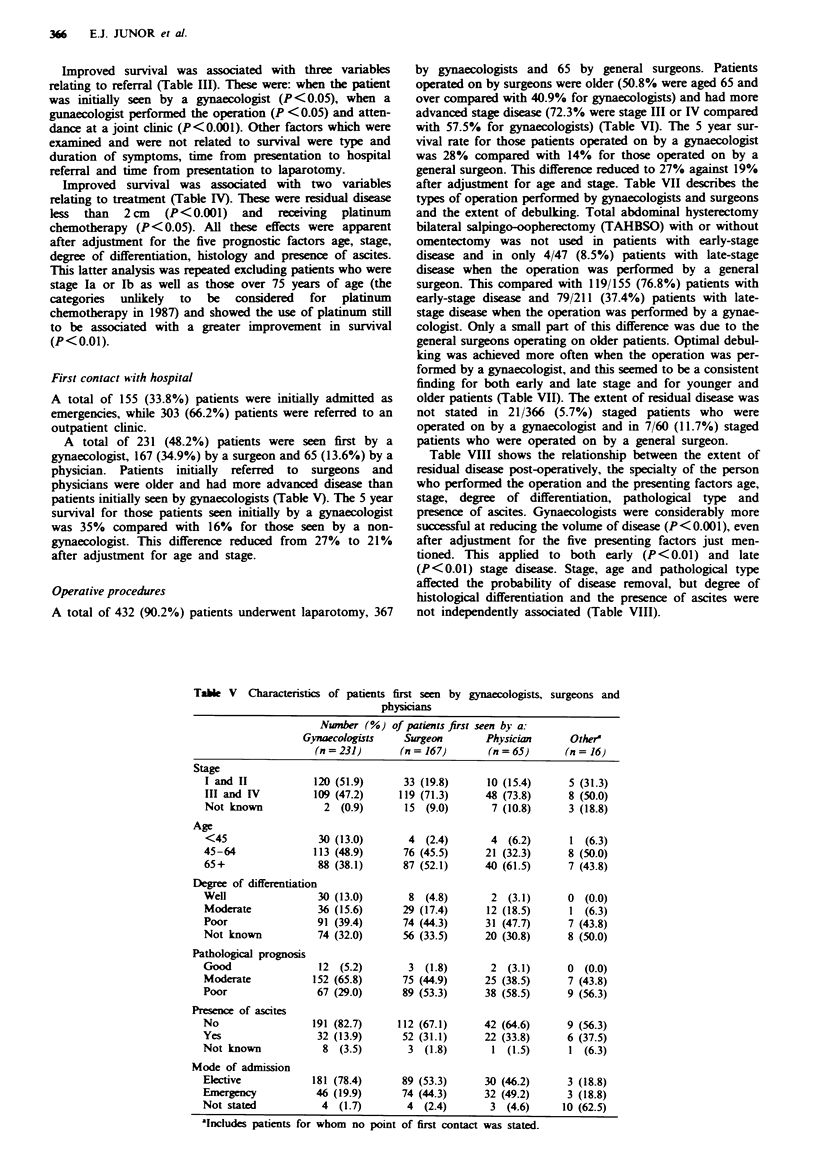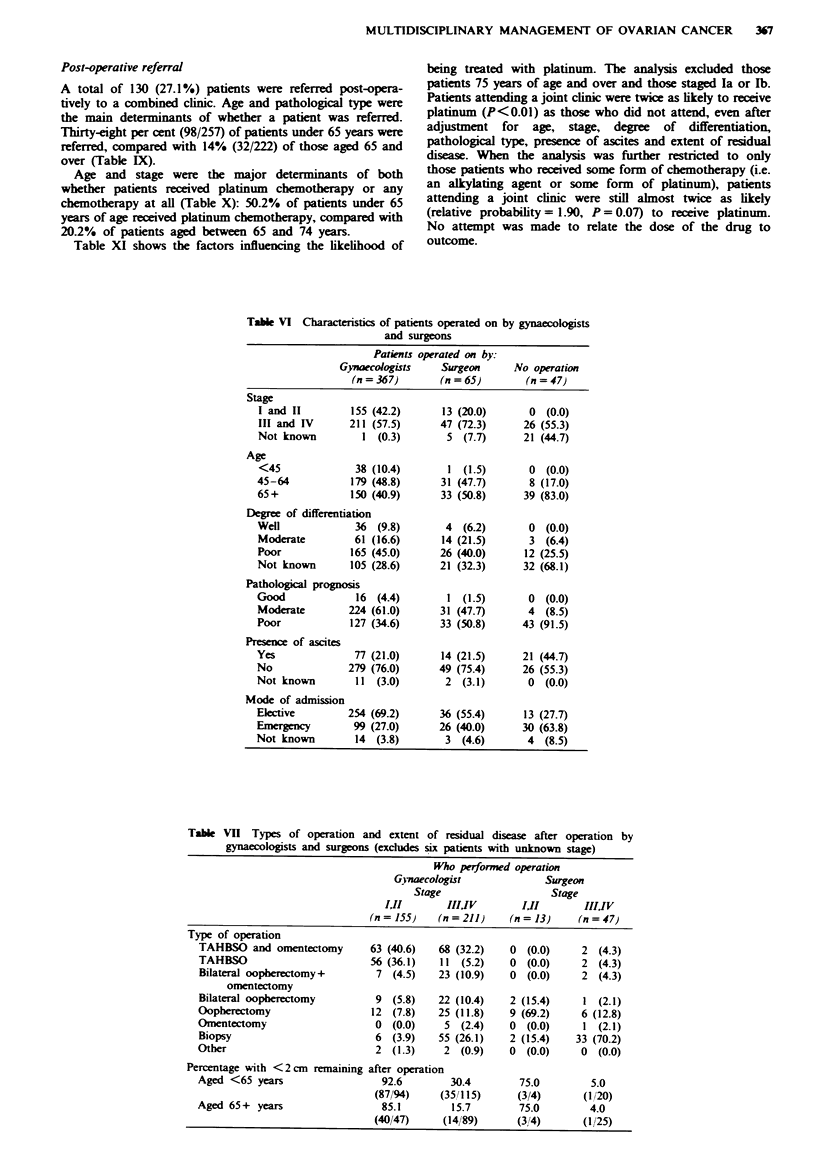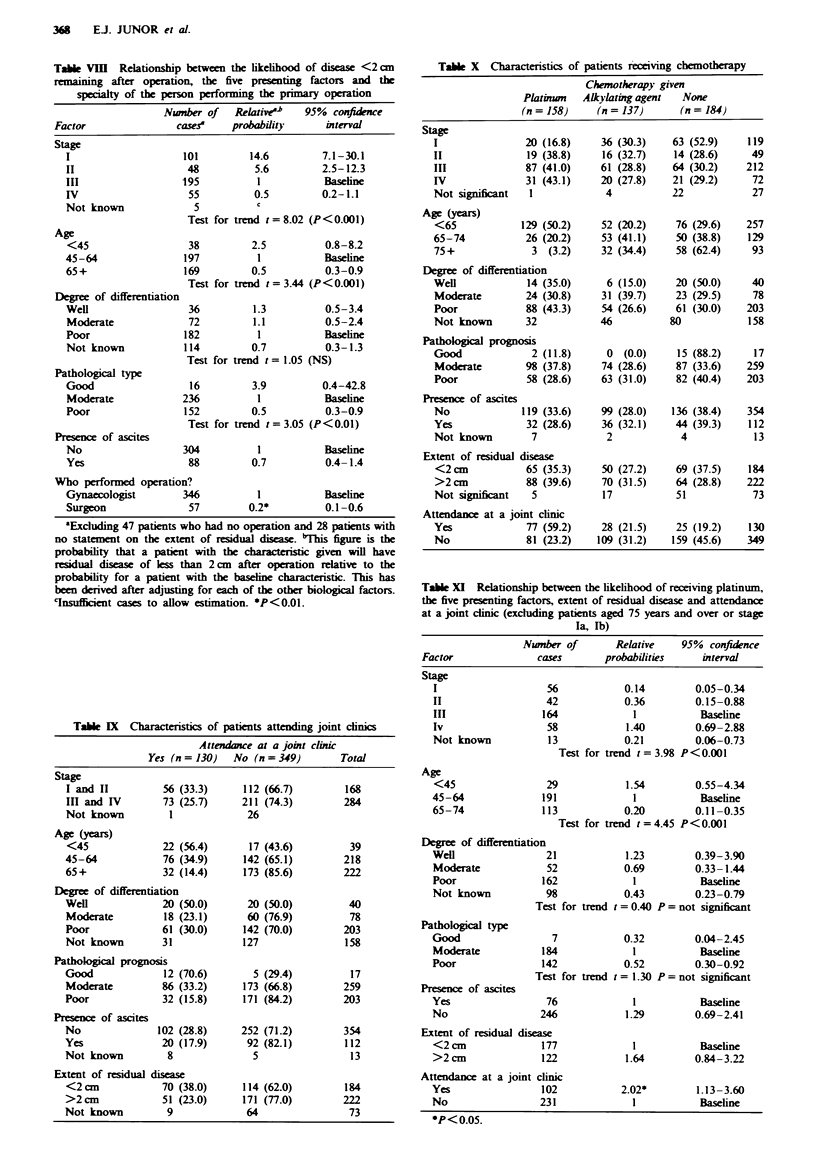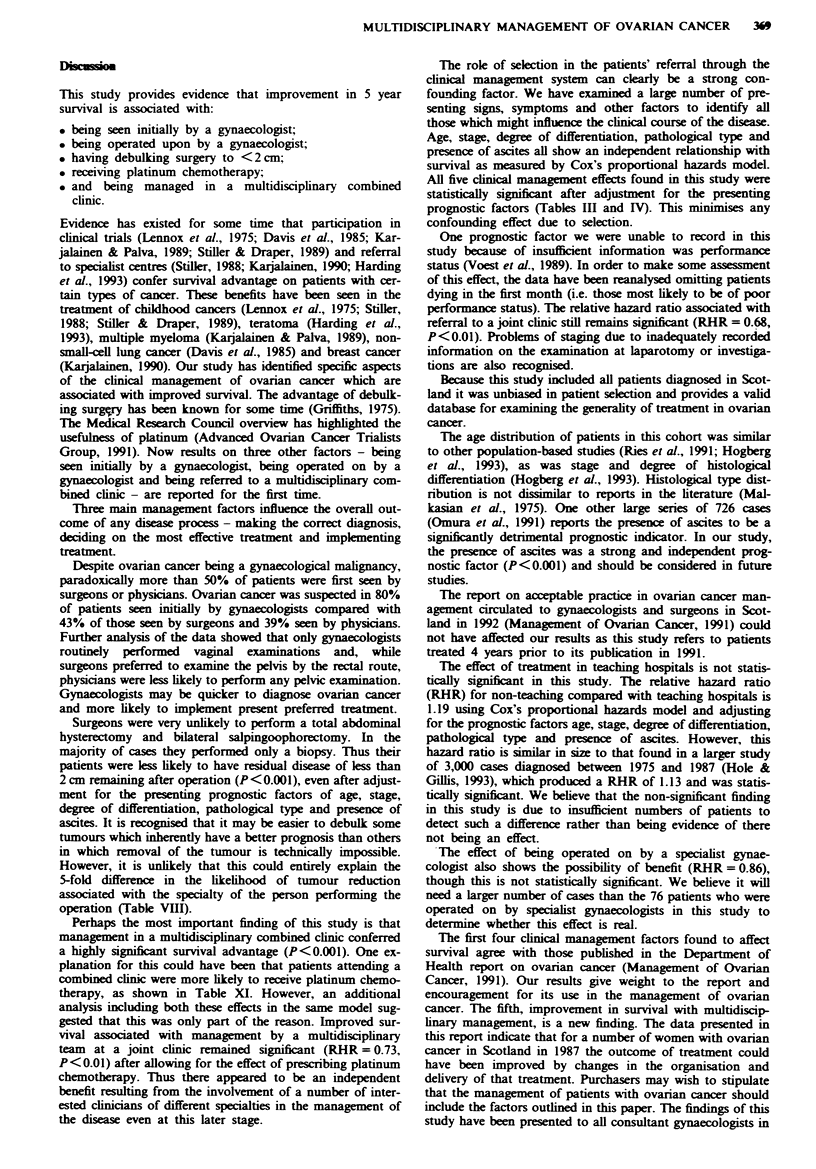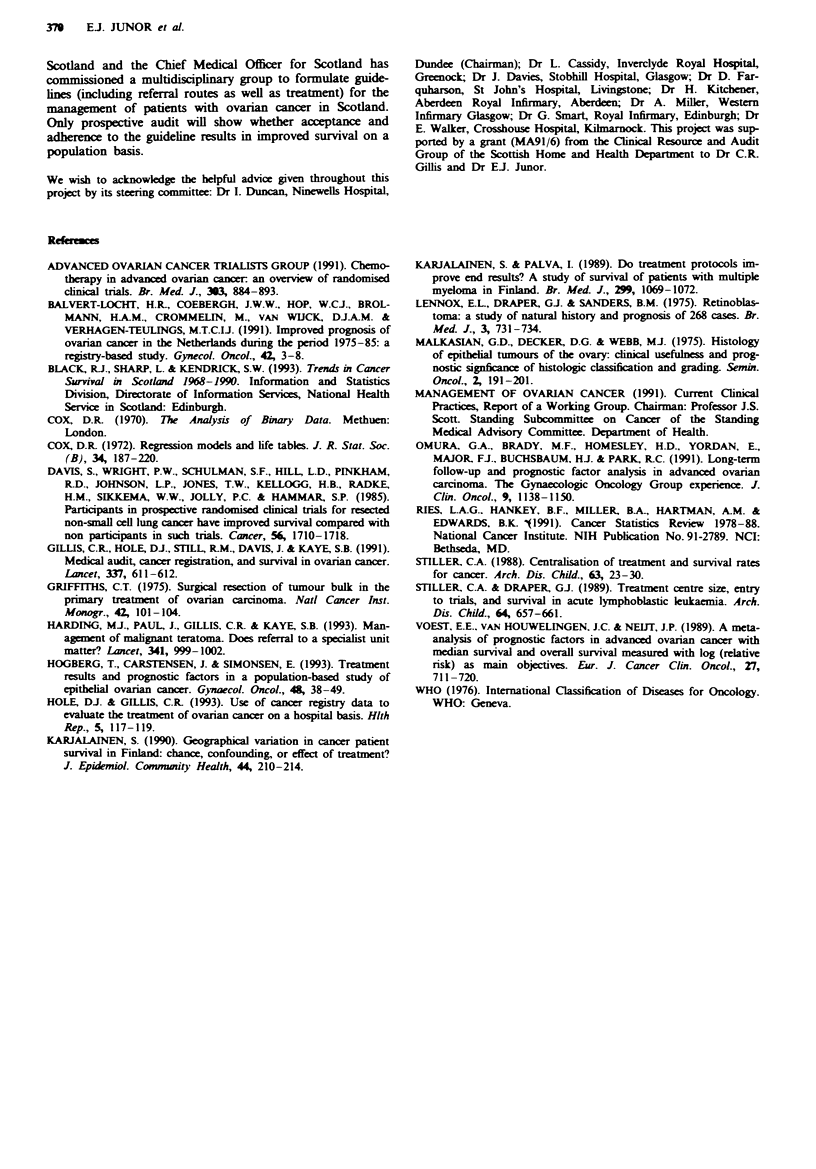# Management of ovarian cancer: referral to a multidisciplinary team matters.

**DOI:** 10.1038/bjc.1994.307

**Published:** 1994-08

**Authors:** E. J. Junor, D. J. Hole, C. R. Gillis

**Affiliations:** West of Scotland Cancer Surveillance Unit, Ruchill Hospital, Glasgow, UK.

## Abstract

Differences in survival outcome for patients with ovarian cancer in Scotland led to an investigation of whether these differences were due to variation in presenting prognostic features or to the organisation and delivery of cancer services. A retrospective study of all 533 cases of ovarian cancer registered in Scotland in 1987 was carried out. After adjustment for age, stage, pathology, degree of differentiation and presence of ascites, survival improved when patients (1) were first seen by a gynaecologist (P < 0.05); (2) were operated on by a gynaecologist (P < 0.05); (3) had residual disease of less than 2 cm post-operatively (P < 0.001); (4) were prescribed platinum chemotherapy (P < 0.05); and (5) were referred to a joint clinic (P < 0.001). When gynaecologists operated the likelihood of smaller residual disease increased (P < 0.001). The improved survival from management by a multidisciplinary team at a joint clinic was not solely due to the prescription of platinum chemotherapy. The results of this study support the contents of the 1991 Department of Health report on present acceptable practice in the management of ovarian cancer, circulated to gynaecologists and surgeons in Scotland in 1992. The new finding that in a common cancer management by a multidisciplinary team at a joint clinic directly affects survival requires urgent attention.


Br. J. Cancer (1994), 70, 363-370 © Macmillan Press Ltd., 1994

### Management of ovarian cancer: referral to a multidisciplinary team matters

E.J. Junor, D.J. Hole & C.R. Gillis

West of Scotland Cancer Surveillance Unit, Ruchill Hospital, Glasgow G20 9NB, UK.

Summary Differences in survival outcome for patients with ovarian cancer in Scotland led to an investigation of whether these differences were due to variation in presenting prognostic features or to the organisation and delivery of cancer services. A retrospective study of all 533 cases of ovarian cancer registered in Scotland in 1987 was carried out. After adjustment for age, stage, pathology, degree of differentiation and presence of ascites, survival improved when patients (1) were first seen by a gynaecologist ($P<0.05$); (2) were operated on by a gynaecologist ($P<0.05$); (3) had residual disease of less than 2 cm post-operatively ($P<0.001$); (4) were prescribed platinum chemotherapy ($P<0.05$); and (5) were referred to a joint clinic ($P<0.001$). When gynaecologists operated the likelihood of smaller residual disease increased ($P<0.001$). The improved survival from management by a multidisciplinary team at a joint clinic was not solely due to the prescription of platinum chemotherapy. The results of this study support the contents of the 1991 Department of Health report on present acceptable practice in the management of ovarian cancer, circulated to gynaecologists and surgeons in Scotland in 1992. The new finding that in a common cancer management by a multidisciplinary team at a joint clinic directly affects survival requires urgent attention.

Survival for patients with ovarian cancer is improving (Balvert-Locht *et al.*, 1991; Ries *et al.*, 1991; Black *et al.*, 1993). In the west of Scotland, 3 year survival for patients under 55 years of age diagnosed between 1975 and 1988 improved from 36% to 50%. Patients aged 55-64 years showed a survival improvement from 23% to 29% over the same time period (Gillis *et al.*, 1991). Patients treated in teaching hospitals appeared to survive longer than those treated elsewhere. These differences were increasing with time (Gillis *et al.*, 1991). However, not all teaching hospitals offered a better outcome nor were all non-teaching hospitals associated with a poorer outcome (Hole & Gillis, 1993).

This analysis (Gillis *et al.*, 1991) was based on cancer registration data, which included age and pathology but no other major prognostic factors. This raised the question of the extent to which other prognostic factors (e.g. stage, degree of differentiation, ascites) or the type of treatment carried out contributed to the differences in survival observed.

A detailed study of all cases of ovarian cancer diagnosed in the Scottish population in 1987 was carried out to identify variations in patient management which might influence survival. The study investigated patients' referral patterns, their treatment and outcome taking account of the above prognostic factors.

### Patients and methods

Each of the 533 patients registered by the Scottish cancer registration scheme with ovarian cancer diagnosed in 1987 was identified. Permission to scrutinise their case records was obtained from their consultants. Thirty-four patients were excluded because of incorrect pathology, year of diagnosis or no information other than a death certificate. The medical records of a further 20 patients could not be found.

Detailed information on presenting features, investigations, pathology, stage, operative procedures, volume of residual disease and subsequent referral and management for the remaining 479 patients (Table I) was abstracted from the case records. Information included details on the specialty of the clinician to whom the patient was referred initially, the specialty of the surgeon performing the initial operation and multidisciplinary management at a joint clinic. Missing or insufficiently detailed information was allocated to a not known category. All patients were flagged with the Registrar General (Scotland) for cause and date of death. All deaths up to 31 December 1992 were included, providing 5 years of follow-up for each patient.

All histological reports were examined by one investigator (E.J.) and coded according to the International Classification of Disease for Oncology (WHO, 1976). No independent histological review was performed.

Staging was performed by one of the authors (E.J.) using the standard FIGO (International Federation of Gynaecology and Obstetrics) classification on the basis of the operation note, pathology report and the results of all available investigations.

Chemotherapy comprised either platinum (cis- or carboplatin), an alkylating agent, a combination of platinum with an alkylating agent or no chemotherapy. For the analysis all patients receiving a platinum drug with or without other agents were called the platinum group; those receiving an alkylating agent alone constituted the alkylating group; the rest made up the no chemotherapy group.

A joint clinic was defined as one in which gynaecologists and oncologists agreed the most appropriate management throughout the entire post-operative treatment.

### Statistical analysis

Cox's proportional hazards model was used to quantify the effect of clinical management on survival, taking account of prognostic factors (Cox, 1972).

The effects of different referral routes were estimated separately from the outcomes of treatment in the following manner. Firstly, a model using only those prognostic variables found to be significant (age, stage, degree of differentiation, histological type and presence of ascites) was fitted (Table II). Secondly, variables relating to patients' referral routes (who first saw them, who operated and attendance at a combined clinic) were added (Table III). Thirdly, treatment variables (amount of residual disease after operation and use of chemotherapy) were considered in addition to those factors included in the first model (Table IV). Factors relating to patients' referral routes were not included in this third model.

The histological types were reduced from 11 to three on

Correspondence: E.J. Junor, Beatson Oncology Centre, Western Infirmary, Glasgow G11 6NT, UK.
Received 4 January 1994; and in revised form 28 March 1994.

the basis of their individual 5 year survival rates in this study. This meant that borderline and germ cell tumours were classified as good; mucinous and serous cystadenocarcinoma and endometrioid, mesonephroid, granulosa cell and miscellaneous tumours were classified as moderate; and adenocarcinoma (no subtype specifically stated) and mixed mesodermal and unknown tumours were classified as poor. Each of the other variables was treated as a categorical variable with a specific category included for missing or unknown information. The category with the largest number of cases was chosen as the baseline in the Cox's proportional hazards analysis.

Comparison of the volume of disease remaining (> or < 2 cm) in relation to the specialty of the surgeon performing the operation while simultaneously adjusting for age, stage, degree of differentiation, pathological type and presence of ascites was carried out using logistic regression (Cox, 1970). A similar approach was used to find what influenced the prescription of platinum chemotherapy. This included using the volume of residual disease in addition to age, stage, degree of differentiation, pathological type and presence of ascites.

### Results

### Survival

Patients' characteristics and unadjusted 5 year survival rates are shown in Table I. The overall 5 year survival rate for the cohort was 23.6% (relative 5 year survival = 27.4%); 62.8% of patients with ovarian cancer presented with advanced disease (stage III or IV) and 63.2% had poorly differentiated tumours. Adenocarcinoma (no subtype specified) was the most common histological type. Each of these prognostic factors was associated with 5 year survival of 14% or less. Ascites was present in 24.0% of patients; 33.8% of patients were admitted as emergency cases. Insufficient or missing information meant that 27 (5.6%) patients could not be staged, 158 (33.0%) patients had no recorded degree of histological differentiation, 15 (3.1%) patients had no recorded histological type and in 13 (2.7%) patients no ascites status was given.

The results of the Cox's proportional hazards analysis relating the risk of death to the five significant prognostic factors is shown in Table II, to the three 'referral' variables (who initially saw the patient, the specialty of the person who performed the operation and whether referral to a joint clinic took place) in Table III and to the two 'treatment' variables (amount of residual disease remaining after operation and the use of platinum) in Table IV. The effects described in Tables III and IV are estimated after adjustment for the five prognostic factors.

The risk of death increased significantly with later stage of presentation ($P<0.001$), increasing age ($P<0.001$), poorer histological differentiation ($P<0.01$), poor pathological type ($P<0.001$) and presence of ascites ($P<0.01$). The risk of death was no greater for patients admitted as emergencies after adjustment for the five prognostic factors just mentioned.

**Table I** Patient characteristics and unadjusted survival for ovarian cancer patients diagnosed in Scotland in 1987

| *Characteristic* | *Number (%)[a] of cases* | | *Percentage surviving 1 year* | *3 years* | *5 years* |
|---|---|---|---|---|---|
| All patients | 479 | | 54 | 30 | 24 |
| Stage | | | | | |
| I | 119 | (26.3) | 92 | 77 | 66 |
| II | 49 | (10.8) | 61 | 41 | 33 |
| III | 212 | (46.9) | 46 | 15 | 8 |
| IV | 72 | (15.9) | 31 | 4 | 0 |
| Not known | 27 | | 4 | 0 | 0 |
| Age group | | | | | |
| <45 | 39 | (8.1) | 82 | 67 | 56 |
| 45–54 | 85 | (17.7) | 67 | 40 | 29 |
| 55–64 | 133 | (27.8) | 61 | 32 | 24 |
| 65–74 | 129 | (26.9) | 51 | 26 | 20 |
| 75+ | 93 | (19.4) | 25 | 12 | 9 |
| Degree of differentiation | | | | | |
| Well | 40 | (12.5) | 70 | 60 | 55 |
| Moderate | 78 | (24.3) | 60 | 35 | 28 |
| Poor | 203 | (63.2) | 50 | 22 | 14 |
| Not known | 158 | | 53 | 32 | 26 |
| Presence of ascites | | | | | |
| No | 354 | (76.0) | 60 | 36 | 29 |
| Yes | 112 | (24.0) | 35 | 12 | 6 |
| Not known | 13 | | 69 | 46 | 23 |
| Histological type | | | | | |
| Borderline | 12 | (2.6) | 100 | 92 | 83 |
| Germ cell | 5 | (1.1) | 100 | 80 | 80 |
| Mucinous adenocarcinoma | 71 | (15.3) | 72 | 52 | 46 |
| Serous adenocarcinoma | 123 | (26.5) | 65 | 34 | 25 |
| Endometrioid | 34 | (7.3) | 59 | 44 | 35 |
| Mesonephroid | 19 | (4.1) | 47 | 37 | 32 |
| Granulosa cell | 7 | (1.5) | 100 | 57 | 57 |
| Adenocarcinoma | 176 | (37.9) | 38 | 13 | 7 |
| Mixed mesodermal | 12 | (2.6) | 25 | 8 | 0 |
| Miscellaneous | 5 | (1.1) | 60 | 40 | 0 |
| Not known | 15 | | 13 | 0 | 0 |
| Mode of admission | | | | | |
| Elective | 303 | (66.2) | 60 | 34 | 26 |
| Emergency | 155 | (33.8) | 44 | 25 | 21 |
| Not known | 21 | | 38 | 24 | 14 |

[a]Percentages have been calculated excluding the 'not knowns'.

**Table II** Relation between prognostic factors and survival amongst ovarian cancer patients diagnosed in 1987 in Scotland

| | *Number of patients* | *Number of deaths in 5 years* | *Relative hazard ratio (RHR)* | *95% confidence interval* |
|---|---|---|---|---|
| Stage | | | | |
| I | 119 | 40 | 0.28 | 0.18–0.40 |
| II | 49 | 33 | 0.77 | 0.53–1.14 |
| III | 212 | 194 | 1 | Baseline |
| IV | 72 | 72 | 1.64 | 1.24–2.16 |
| Not known | 27 | 27 | 1.29 | 0.88–1.90 |
| | | Test for trend $t=6.38$ ($P<0.001$) | | |
| Age group | | | | |
| <45 | 39 | 17 | 0.65 | 0.38–1.11 |
| 45–54 | 85 | 60 | 0.85 | 0.62–1.18 |
| 55–64 | 133 | 101 | 1 | Baseline |
| 65–74 | 129 | 103 | 1.17 | 0.88–1.55 |
| 75+ | 93 | 85 | 2.02 | 1.50–2.72 |
| | | Test for trend $t=5.37$ ($P<0.001$) | | |
| Degree of differentiation | | | | |
| Well | 40 | 18 | 0.50 | 0.30–0.82 |
| Moderate | 78 | 56 | 0.75 | 0.55–1.02 |
| Poor | 203 | 175 | 1 | Baseline |
| Not known | 158 | 117 | 0.95 | 0.75–1.22 |
| | | Test for trend $t=3.02$ ($P<0.01$) | | |
| Pathological prognosis | | | | |
| Good | 17 | 3 | 0.34 | 0.10–1.10 |
| Moderate | 259 | 173 | 1 | Baseline |
| Poor | 203 | 190 | 1.61 | 1.28–2.02 |
| | | Test for trend $t=4.56$ ($P<0.001$) | | |
| Ascites | | | | |
| No | 354 | 251 | 1 | Baseline |
| Yes | 112 | 105 | 1.56* | 1.23–1.98 |

*$P<0.01$.

**Table III** Influence of 'referral' factors on survival after adjustment for the five biological factors shown in Table II

| | *Number of patients* | *Number of deaths in 5 years* | *Relative hazard ratio* | *95% confidence interval* |
|---|---|---|---|---|
| Who first saw patient? | | | | |
| Gynaecologist | 231 | 150 | 1 | Baseline |
| Non-gynaecologist | 248 | 216 | 1.34* | 1.05–1.70 |
| Who performed operation? | | | | |
| Gynaecologist | 367 | 263 | 1 | Baseline |
| Surgeon | 65 | 56 | 1.37* | 1.05–1.77 |
| Attendance at combined clinic | | | | |
| Yes | 130 | 84 | 0.60** | 0.46–0.78 |
| No | 349 | 282 | 1 | Baseline |

*$P<0.05$; **$P<0.001$.

**Table IV** Relationship of 'treatment' factors on survival after adjustment for the five biological factors shown in Table II

| | *Number of patients* | *Number of deaths in 5 years* | *Relative hazard ratio* | *95% confidence interval* |
|---|---|---|---|---|
| Residual disease | | | | |
| <2 cm | 184 | 89 | 0.50** | 0.37–0.66 |
| >2 cm | 222 | 214 | 1 | Baseline |
| Use of chemotherapeutic drugs | | | | |
| Platinum | 158 | 128 | 0.72* | 0.53–0.97 |
| Alkylating | 137 | 103 | 1 | Baseline |
| No chemotherapy | 184 | 135 | 1.74** | 1.33–2.29 |

*$P<0.05$; **$P<0.001$.

Improved survival was associated with three variables relating to referral (Table III). These were: when the patient was initially seen by a gynaecologist ($P<0.05$), when a gunaecologist performed the operation ($P<0.05$) and attendance at a joint clinic ($P<0.001$). Other factors which were examined and were not related to survival were type and duration of symptoms, time from presentation to hospital referral and time from presentation to laparotomy.

Improved survival was associated with two variables relating to treatment (Table IV). These were residual disease less than 2 cm ($P<0.001$) and receiving platinum chemotherapy ($P<0.05$). All these effects were apparent after adjustment for the five prognostic factors age, stage, degree of differentiation, histology and presence of ascites. This latter analysis was repeated excluding patients who were stage Ia or Ib as well as those over 75 years of age (the categories unlikely to be considered for platinum chemotherapy in 1987) and showed the use of platinum still to be associated with a greater improvement in survival ($P<0.01$).

*First contact with hospital*

A total of 155 (33.8%) patients were initially admitted as emergencies, while 303 (66.2%) patients were referred to an outpatient clinic.

A total of 231 (48.2%) patients were seen first by a gynaecologist, 167 (34.9%) by a surgeon and 65 (13.6%) by a physician. Patients initially referred to surgeons and physicians were older and had more advanced disease than patients initially seen by gynaecologists (Table V). The 5 year survival for those patients seen initially by a gynaecologist was 35% compared with 16% for those seen by a non-gynaecologist. This difference reduced from 27% to 21% after adjustment for age and stage.

*Operative procedures*

A total of 432 (90.2%) patients underwent laparotomy, 367 by gynaecologists and 65 by general surgeons. Patients operated on by surgeons were older (50.8% were aged 65 and over compared with 40.9% for gynaecologists) and had more advanced stage disease (72.3% were stage III or IV compared with 57.5% for gynaecologists) (Table VI). The 5 year survival rate for those patients operated on by a gynaecologist was 28% compared with 14% for those operated on by a general surgeon. This difference reduced to 27% against 19% after adjustment for age and stage. Table VII describes the types of operation performed by gynaecologists and surgeons and the extent of debulking. Total abdominal hysterectomy bilateral salpingo-oopherectomy (TAHBSO) with or without omentectomy was not used in patients with early-stage disease and in only 4/47 (8.5%) patients with late-stage disease when the operation was performed by a general surgeon. This compared with 119/155 (76.8%) patients with early-stage disease and 79/211 (37.4%) patients with late-stage disease when the operation was performed by a gynaecologist. Only a small part of this difference was due to the general surgeons operating on older patients. Optimal debulking was achieved more often when the operation was performed by a gynaecologist, and this seemed to be a consistent finding for both early and late stage and for younger and older patients (Table VII). The extent of residual disease was not stated in 21/366 (5.7%) staged patients who were operated on by a gynaecologist and in 7/60 (11.7%) staged patients who were operated on by a general surgeon.

Table VIII shows the relationship between the extent of residual disease post-operatively, the specialty of the person who performed the operation and the presenting factors age, stage, degree of differentiation, pathological type and presence of ascites. Gynaecologists were considerably more successful at reducing the volume of disease ($P<0.001$), even after adjustment for the five presenting factors just mentioned. This applied to both early ($P<0.01$) and late ($P<0.01$) stage disease. Stage, age and pathological type affected the probability of disease removal, but degree of histological differentiation and the presence of ascites were not independently associated (Table VIII).

**Table V** Characteristics of patients first seen by gynaecologists, surgeons and physicians

| | *Number (%) of patients first seen by a:* Gynaecologists (n = 231) | Surgeon (n = 167) | Physician (n = 65) | Other[a] (n = 16) |
|---|---|---|---|---|
| Stage | | | | |
| I and II | 120 (51.9) | 33 (19.8) | 10 (15.4) | 5 (31.3) |
| III and IV | 109 (47.2) | 119 (71.3) | 48 (73.8) | 8 (50.0) |
| Not known | 2 (0.9) | 15 (9.0) | 7 (10.8) | 3 (18.8) |
| Age | | | | |
| <45 | 30 (13.0) | 4 (2.4) | 4 (6.2) | 1 (6.3) |
| 45–64 | 113 (48.9) | 76 (45.5) | 21 (32.3) | 8 (50.0) |
| 65+ | 88 (38.1) | 87 (52.1) | 40 (61.5) | 7 (43.8) |
| Degree of differentiation | | | | |
| Well | 30 (13.0) | 8 (4.8) | 2 (3.1) | 0 (0.0) |
| Moderate | 36 (15.6) | 29 (17.4) | 12 (18.5) | 1 (6.3) |
| Poor | 91 (39.4) | 74 (44.3) | 31 (47.7) | 7 (43.8) |
| Not known | 74 (32.0) | 56 (33.5) | 20 (30.8) | 8 (50.0) |
| Pathological prognosis | | | | |
| Good | 12 (5.2) | 3 (1.8) | 2 (3.1) | 0 (0.0) |
| Moderate | 152 (65.8) | 75 (44.9) | 25 (38.5) | 7 (43.8) |
| Poor | 67 (29.0) | 89 (53.3) | 38 (58.5) | 9 (56.3) |
| Presence of ascites | | | | |
| No | 191 (82.7) | 112 (67.1) | 42 (64.6) | 9 (56.3) |
| Yes | 32 (13.9) | 52 (31.1) | 22 (33.8) | 6 (37.5) |
| Not known | 8 (3.5) | 3 (1.8) | 1 (1.5) | 1 (6.3) |
| Mode of admission | | | | |
| Elective | 181 (78.4) | 89 (53.3) | 30 (46.2) | 3 (18.8) |
| Emergency | 46 (19.9) | 74 (44.3) | 32 (49.2) | 3 (18.8) |
| Not stated | 4 (1.7) | 4 (2.4) | 3 (4.6) | 10 (62.5) |

[a]Includes patients for whom no point of first contact was stated.

*Post-operative referral*

A total of 130 (27.1%) patients were referred post-operatively to a combined clinic. Age and pathological type were the main determinants of whether a patient was referred. Thirty-eight per cent (98/257) of patients under 65 years were referred, compared with 14% (32/222) of those aged 65 and over (Table IX).

Age and stage were the major determinants of both whether patients received platinum chemotherapy or any chemotherapy at all (Table X): 50.2% of patients under 65 years of age received platinum chemotherapy, compared with 20.2% of patients aged between 65 and 74 years.

Table XI shows the factors influencing the likelihood of being treated with platinum. The analysis excluded those patients 75 years of age and over and those staged Ia or Ib. Patients attending a joint clinic were twice as likely to receive platinum ($P<0.01$) as those who did not attend, even after adjustment for age, stage, degree of differentiation, pathological type, presence of ascites and extent of residual disease. When the analysis was further restricted to only those patients who received some form of chemotherapy (i.e. an alkylating agent or some form of platinum), patients attending a joint clinic were still almost twice as likely (relative probability = 1.90, $P=0.07$) to receive platinum. No attempt was made to relate the dose of the drug to outcome.

**Table VI** Characteristics of patients operated on by gynaecologists and surgeons

| | *Patients operated on by:* Gynaecologists ($n=367$) | Surgeon ($n=65$) | No operation ($n=47$) |
|---|---|---|---|
| Stage | | | |
| I and II | 155 (42.2) | 13 (20.0) | 0 (0.0) |
| III and IV | 211 (57.5) | 47 (72.3) | 26 (55.3) |
| Not known | 1 (0.3) | 5 (7.7) | 21 (44.7) |
| Age | | | |
| <45 | 38 (10.4) | 1 (1.5) | 0 (0.0) |
| 45–64 | 179 (48.8) | 31 (47.7) | 8 (17.0) |
| 65+ | 150 (40.9) | 33 (50.8) | 39 (83.0) |
| Degree of differentiation | | | |
| Well | 36 (9.8) | 4 (6.2) | 0 (0.0) |
| Moderate | 61 (16.6) | 14 (21.5) | 3 (6.4) |
| Poor | 165 (45.0) | 26 (40.0) | 12 (25.5) |
| Not known | 105 (28.6) | 21 (32.3) | 32 (68.1) |
| Pathological prognosis | | | |
| Good | 16 (4.4) | 1 (1.5) | 0 (0.0) |
| Moderate | 224 (61.0) | 31 (47.7) | 4 (8.5) |
| Poor | 127 (34.6) | 33 (50.8) | 43 (91.5) |
| Presence of ascites | | | |
| Yes | 77 (21.0) | 14 (21.5) | 21 (44.7) |
| No | 279 (76.0) | 49 (75.4) | 26 (55.3) |
| Not known | 11 (3.0) | 2 (3.1) | 0 (0.0) |
| Mode of admission | | | |
| Elective | 254 (69.2) | 36 (55.4) | 13 (27.7) |
| Emergency | 99 (27.0) | 26 (40.0) | 30 (63.8) |
| Not known | 14 (3.8) | 3 (4.6) | 4 (8.5) |

**Table VII** Types of operation and extent of residual disease after operation by gynaecologists and surgeons (excludes six patients with unknown stage)

| | *Who performed operation* Gynaecologist Stage I,II ($n=155$) | Gynaecologist Stage III,IV ($n=211$) | Surgeon Stage I,II ($n=13$) | Surgeon Stage III,IV ($n=47$) |
|---|---|---|---|---|
| Type of operation | | | | |
| TAHBSO and omentectomy | 63 (40.6) | 68 (32.2) | 0 (0.0) | 2 (4.3) |
| TAHBSO | 56 (36.1) | 11 (5.2) | 0 (0.0) | 2 (4.3) |
| Bilateral oopherectomy + omentectomy | 7 (4.5) | 23 (10.9) | 0 (0.0) | 2 (4.3) |
| Bilateral oopherectomy | 9 (5.8) | 22 (10.4) | 2 (15.4) | 1 (2.1) |
| Oopherectomy | 12 (7.8) | 25 (11.8) | 9 (69.2) | 6 (12.8) |
| Omentectomy | 0 (0.0) | 5 (2.4) | 0 (0.0) | 1 (2.1) |
| Biopsy | 6 (3.9) | 55 (26.1) | 2 (15.4) | 33 (70.2) |
| Other | 2 (1.3) | 2 (0.9) | 0 (0.0) | 0 (0.0) |
| Percentage with <2 cm remaining after operation | | | | |
| Aged <65 years | 92.6 (87/94) | 30.4 (35/115) | 75.0 (3/4) | 5.0 (1/20) |
| Aged 65+ years | 85.1 (40/47) | 15.7 (14/89) | 75.0 (3/4) | 4.0 (1/25) |

**Table VIII** Relationship between the likelihood of disease <2 cm remaining after operation, the five presenting factors and the specialty of the person performing the primary operation

| Factor | Number of cases[a] | Relative[a,b] probability | 95% confidence interval |
|---|---|---|---|
| Stage | | | |
| I | 101 | 14.6 | 7.1–30.1 |
| II | 48 | 5.6 | 2.5–12.3 |
| III | 195 | 1 | Baseline |
| IV | 55 | 0.5 | 0.2–1.1 |
| Not known | 5 | [c] | |
| | Test for trend $t = 8.02$ ($P<0.001$) | | |
| Age | | | |
| <45 | 38 | 2.5 | 0.8–8.2 |
| 45–64 | 197 | 1 | Baseline |
| 65+ | 169 | 0.5 | 0.3–0.9 |
| | Test for trend $t = 3.44$ ($P<0.001$) | | |
| Degree of differentiation | | | |
| Well | 36 | 1.3 | 0.5–3.4 |
| Moderate | 72 | 1.1 | 0.5–2.4 |
| Poor | 182 | 1 | Baseline |
| Not known | 114 | 0.7 | 0.3–1.3 |
| | Test for trend $t = 1.05$ (NS) | | |
| Pathological type | | | |
| Good | 16 | 3.9 | 0.4–42.8 |
| Moderate | 236 | 1 | Baseline |
| Poor | 152 | 0.5 | 0.3–0.9 |
| | Test for trend $t = 3.05$ ($P<0.01$) | | |
| Presence of ascites | | | |
| No | 304 | 1 | Baseline |
| Yes | 88 | 0.7 | 0.4–1.4 |
| Who performed operation? | | | |
| Gynaecologist | 346 | 1 | Baseline |
| Surgeon | 57 | 0.2* | 0.1–0.6 |

[a]Excluding 47 patients who had no operation and 28 patients with no statement on the extent of residual disease. [b]This figure is the probability that a patient with the characteristic given will have residual disease of less than 2 cm after operation relative to the probability for a patient with the baseline characteristic. This has been derived after adjusting for each of the other biological factors. [c]Insufficient cases to allow estimation. *$P<0.01$.

**Table IX** Characteristics of patients attending joint clinics

| | Attendance at a joint clinic | | |
|---|---|---|---|
| | Yes ($n = 130$) | No ($n = 349$) | Total |
| Stage | | | |
| I and II | 56 (33.3) | 112 (66.7) | 168 |
| III and IV | 73 (25.7) | 211 (74.3) | 284 |
| Not known | 1 | 26 | |
| Age (years) | | | |
| <45 | 22 (56.4) | 17 (43.6) | 39 |
| 45–64 | 76 (34.9) | 142 (65.1) | 218 |
| 65+ | 32 (14.4) | 173 (85.6) | 222 |
| Degree of differentiation | | | |
| Well | 20 (50.0) | 20 (50.0) | 40 |
| Moderate | 18 (23.1) | 60 (76.9) | 78 |
| Poor | 61 (30.0) | 142 (70.0) | 203 |
| Not known | 31 | 127 | 158 |
| Pathological prognosis | | | |
| Good | 12 (70.6) | 5 (29.4) | 17 |
| Moderate | 86 (33.2) | 173 (66.8) | 259 |
| Poor | 32 (15.8) | 171 (84.2) | 203 |
| Presence of ascites | | | |
| No | 102 (28.8) | 252 (71.2) | 354 |
| Yes | 20 (17.9) | 92 (82.1) | 112 |
| Not known | 8 | 5 | 13 |
| Extent of residual disease | | | |
| <2 cm | 70 (38.0) | 114 (62.0) | 184 |
| >2 cm | 51 (23.0) | 171 (77.0) | 222 |
| Not known | 9 | 64 | 73 |

**Table X** Characteristics of patients receiving chemotherapy

| | Chemotherapy given | | | |
|---|---|---|---|---|
| | Platinum ($n = 158$) | Alkylating agent ($n = 137$) | None ($n = 184$) | |
| Stage | | | | |
| I | 20 (16.8) | 36 (30.3) | 63 (52.9) | 119 |
| II | 19 (38.8) | 16 (32.7) | 14 (28.6) | 49 |
| III | 87 (41.0) | 61 (28.8) | 64 (30.2) | 212 |
| IV | 31 (43.1) | 20 (27.8) | 21 (29.2) | 72 |
| Not significant | 1 | 4 | 22 | 27 |
| Age (years) | | | | |
| <65 | 129 (50.2) | 52 (20.2) | 76 (29.6) | 257 |
| 65–74 | 26 (20.2) | 53 (41.1) | 50 (38.8) | 129 |
| 75+ | 3 (3.2) | 32 (34.4) | 58 (62.4) | 93 |
| Degree of differentiation | | | | |
| Well | 14 (35.0) | 6 (15.0) | 20 (50.0) | 40 |
| Moderate | 24 (30.8) | 31 (39.7) | 23 (29.5) | 78 |
| Poor | 88 (43.3) | 54 (26.6) | 61 (30.0) | 203 |
| Not known | 32 | 46 | 80 | 158 |
| Pathological prognosis | | | | |
| Good | 2 (11.8) | 0 (0.0) | 15 (88.2) | 17 |
| Moderate | 98 (37.8) | 74 (28.6) | 87 (33.6) | 259 |
| Poor | 58 (28.6) | 63 (31.0) | 82 (40.4) | 203 |
| Presence of ascites | | | | |
| No | 119 (33.6) | 99 (28.0) | 136 (38.4) | 354 |
| Yes | 32 (28.6) | 36 (32.1) | 44 (39.3) | 112 |
| Not known | 7 | 2 | 4 | 13 |
| Extent of residual disease | | | | |
| <2 cm | 65 (35.3) | 50 (27.2) | 69 (37.5) | 184 |
| >2 cm | 88 (39.6) | 70 (31.5) | 64 (28.8) | 222 |
| Not significant | 5 | 17 | 51 | 73 |
| Attendance at a joint clinic | | | | |
| Yes | 77 (59.2) | 28 (21.5) | 25 (19.2) | 130 |
| No | 81 (23.2) | 109 (31.2) | 159 (45.6) | 349 |

**Table XI** Relationship between the likelihood of receiving platinum, the five presenting factors, extent of residual disease and attendance at a joint clinic (excluding patients aged 75 years and over or stage Ia, Ib)

| Factor | Number of cases | Relative probabilities | 95% confidence interval |
|---|---|---|---|
| Stage | | | |
| I | 56 | 0.14 | 0.05–0.34 |
| II | 42 | 0.36 | 0.15–0.88 |
| III | 164 | 1 | Baseline |
| Iv | 58 | 1.40 | 0.69–2.88 |
| Not known | 13 | 0.21 | 0.06–0.73 |
| | Test for trend $t = 3.98$ $P<0.001$ | | |
| Age | | | |
| <45 | 29 | 1.54 | 0.55–4.34 |
| 45–64 | 191 | 1 | Baseline |
| 65–74 | 113 | 0.20 | 0.11–0.35 |
| | Test for trend $t = 4.45$ $P<0.001$ | | |
| Degree of differentiation | | | |
| Well | 21 | 1.23 | 0.39–3.90 |
| Moderate | 52 | 0.69 | 0.33–1.44 |
| Poor | 162 | 1 | Baseline |
| Not known | 98 | 0.43 | 0.23–0.79 |
| | Test for trend $t = 0.40$ $P =$ not significant | | |
| Pathological type | | | |
| Good | 7 | 0.32 | 0.04–2.45 |
| Moderate | 184 | 1 | Baseline |
| Poor | 142 | 0.52 | 0.30–0.92 |
| | Test for trend $t = 1.30$ $P =$ not significant | | |
| Presence of ascites | | | |
| Yes | 76 | 1 | Baseline |
| No | 246 | 1.29 | 0.69–2.41 |
| Extent of residual disease | | | |
| <2 cm | 177 | 1 | Baseline |
| >2 cm | 122 | 1.64 | 0.84–3.22 |
| Attendance at a joint clinic | | | |
| Yes | 102 | 2.02* | 1.13–3.60 |
| No | 231 | 1 | Baseline |

*$P<0.05$.

### Discussion

This study provides evidence that improvement in 5 year survival is associated with:

- being seen initially by a gynaecologist;
- being operated upon by a gynaecologist;
- having debulking surgery to $<2$ cm;
- receiving platinum chemotherapy;
- and being managed in a multidisciplinary combined clinic.

Evidence has existed for some time that participation in clinical trials (Lennox *et al.*, 1975; Davis *et al.*, 1985; Karjalainen & Palva, 1989; Stiller & Draper, 1989) and referral to specialist centres (Stiller, 1988; Karjalainen, 1990; Harding *et al.*, 1993) confer survival advantage on patients with certain types of cancer. These benefits have been seen in the treatment of childhood cancers (Lennox *et al.*, 1975; Stiller, 1988; Stiller & Draper, 1989), teratoma (Harding *et al.*, 1993), multiple myeloma (Karjalainen & Palva, 1989), non-small-cell lung cancer (Davis *et al.*, 1985) and breast cancer (Karjalainen, 1990). Our study has identified specific aspects of the clinical management of ovarian cancer which are associated with improved survival. The advantage of debulking surgery has been known for some time (Griffiths, 1975). The Medical Research Council overview has highlighted the usefulness of platinum (Advanced Ovarian Cancer Trialists Group, 1991). Now results on three other factors – being seen initially by a gynaecologist, being operated on by a gynaecologist and being referred to a multidisciplinary combined clinic – are reported for the first time.

Three main management factors influence the overall outcome of any disease process – making the correct diagnosis, deciding on the most effective treatment and implementing treatment.

Despite ovarian cancer being a gynaecological malignancy, paradoxically more than 50% of patients were first seen by surgeons or physicians. Ovarian cancer was suspected in 80% of patients seen initially by gynaecologists compared with 43% of those seen by surgeons and 39% seen by physicians. Further analysis of the data showed that only gynaecologists routinely performed vaginal examinations and, while surgeons preferred to examine the pelvis by the rectal route, physicians were less likely to perform any pelvic examination. Gynaecologists may be quicker to diagnose ovarian cancer and more likely to implement present preferred treatment.

Surgeons were very unlikely to perform a total abdominal hysterectomy and bilateral salpingoophorectomy. In the majority of cases they performed only a biopsy. Thus their patients were less likely to have residual disease of less than 2 cm remaining after operation ($P<0.001$), even after adjustment for the presenting prognostic factors of age, stage, degree of differentiation, pathological type and presence of ascites. It is recognised that it may be easier to debulk some tumours which inherently have a better prognosis than others in which removal of the tumour is technically impossible. However, it is unlikely that this could entirely explain the 5-fold difference in the likelihood of tumour reduction associated with the specialty of the person performing the operation (Table VIII).

Perhaps the most important finding of this study is that management in a multidisciplinary combined clinic conferred a highly significant survival advantage ($P<0.001$). One explanation for this could have been that patients attending a combined clinic were more likely to receive platinum chemotherapy, as shown in Table XI. However, an additional analysis including both these effects in the same model suggested that this was only part of the reason. Improved survival associated with management by a multidisciplinary team at a joint clinic remained significant (RHR = 0.73, $P<0.01$) after allowing for the effect of prescribing platinum chemotherapy. Thus there appeared to be an independent benefit resulting from the involvement of a number of interested clinicians of different specialties in the management of the disease even at this later stage.

The role of selection in the patients' referral through the clinical management system can clearly be a strong confounding factor. We have examined a large number of presenting signs, symptoms and other factors to identify all those which might influence the clinical course of the disease. Age, stage, degree of differentiation, pathological type and presence of ascites all show an independent relationship with survival as measured by Cox's proportional hazards model. All five clinical management effects found in this study were statistically significant after adjustment for the presenting prognostic factors (Tables III and IV). This minimises any confounding effect due to selection.

One prognostic factor we were unable to record in this study because of insufficient information was performance status (Voest *et al.*, 1989). In order to make some assessment of this effect, the data have been reanalysed omitting patients dying in the first month (i.e. those most likely to be of poor performance status). The relative hazard ratio associated with referral to a joint clinic still remains significant (RHR = 0.68, $P<0.01$). Problems of staging due to inadequately recorded information on the examination at laparotomy or investigations are also recognised.

Because this study included all patients diagnosed in Scotland it was unbiased in patient selection and provides a valid database for examining the generality of treatment in ovarian cancer.

The age distribution of patients in this cohort was similar to other population-based studies (Ries *et al.*, 1991; Hogberg *et al.*, 1993), as was stage and degree of histological differentiation (Hogberg *et al.*, 1993). Histological type distribution is not dissimilar to reports in the literature (Malkasian *et al.*, 1975). One other large series of 726 cases (Omura *et al.*, 1991) reports the presence of ascites to be a significantly detrimental prognostic indicator. In our study, the presence of ascites was a strong and independent prognostic factor ($P<0.001$) and should be considered in future studies.

The report on acceptable practice in ovarian cancer management circulated to gynaecologists and surgeons in Scotland in 1992 (Management of Ovarian Cancer, 1991) could not have affected our results as this study refers to patients treated 4 years prior to its publication in 1991.

The effect of treatment in teaching hospitals is not statistically significant in this study. The relative hazard ratio (RHR) for non-teaching compared with teaching hospitals is 1.19 using Cox's proportional hazards model and adjusting for the prognostic factors age, stage, degree of differentiation, pathological type and presence of ascites. However, this hazard ratio is similar in size to that found in a larger study of 3,000 cases diagnosed between 1975 and 1987 (Hole & Gillis, 1993), which produced a RHR of 1.13 and was statistically significant. We believe that the non-significant finding in this study is due to insufficient numbers of patients to detect such a difference rather than being evidence of there not being an effect.

The effect of being operated on by a specialist gynaecologist also shows the possibility of benefit (RHR = 0.86), though this is not statistically significant. We believe it will need a larger number of cases than the 76 patients who were operated on by specialist gynaecologists in this study to determine whether this effect is real.

The first four clinical management factors found to affect survival agree with those published in the Department of Health report on ovarian cancer (Management of Ovarian Cancer, 1991). Our results give weight to the report and encouragement for its use in the management of ovarian cancer. The fifth, improvement in survival with multidisciplinary management, is a new finding. The data presented in this report indicate that for a number of women with ovarian cancer in Scotland in 1987 the outcome of treatment could have been improved by changes in the organisation and delivery of that treatment. Purchasers may wish to stipulate that the management of patients with ovarian cancer should include the factors outlined in this paper. The findings of this study have been presented to all consultant gynaecologists in

Scotland and the Chief Medical Officer for Scotland has commissioned a multidisciplinary group to formulate guidelines (including referral routes as well as treatment) for the management of patients with ovarian cancer in Scotland. Only prospective audit will show whether acceptance and adherence to the guideline results in improved survival on a population basis.

We wish to acknowledge the helpful advice given throughout this project by its steering committee: Dr I. Duncan, Ninewells Hospital, Dundee (Chairman); Dr L. Cassidy, Inverclyde Royal Hospital, Greenock; Dr J. Davies, Stobhill Hospital, Glasgow; Dr D. Farquharson, St John's Hospital, Livingstone; Dr H. Kitchener, Aberdeen Royal Infirmary, Aberdeen; Dr A. Miller, Western Infirmary Glasgow; Dr G. Smart, Royal Infirmary, Edinburgh; Dr E. Walker, Crosshouse Hospital, Kilmarnock. This project was supported by a grant (MA91/6) from the Clinical Resource and Audit Group of the Scottish Home and Health Department to Dr C.R. Gillis and Dr E.J. Junor.

### References

ADVANCED OVARIAN CANCER TRIALISTS GROUP (1991). Chemotherapy in advanced ovarian cancer: an overview of randomised clinical trials. *Br. Med. J.*, **303**, 884-893.

BALVERT-LOCHT, H.R., COEBERGH, J.W.W., HOP, W.C.J., BROLMANN, H.A.M., CROMMELIN, M., VAN WIJCK, D.J.A.M. & VERHAGEN-TEULINGS, M.T.C.I.J. (1991). Improved prognosis of ovarian cancer in the Netherlands during the period 1975-85: a registry-based study. *Gynecol. Oncol.*, **42**, 3-8.

BLACK, R.J., SHARP, L. & KENDRICK, S.W. (1993). *Trends in Cancer Survival in Scotland 1968-1990*. Information and Statistics Division, Directorate of Information Services, National Health Service in Scotland: Edinburgh.

COX, D.R. (1970). *The Analysis of Binary Data*. Methuen: London.

COX, D.R. (1972). Regression models and life tables. *J. R. Stat. Soc. (B)*, **34**, 187-220.

DAVIS, S., WRIGHT, P.W., SCHULMAN, S.F., HILL, L.D., PINKHAM, R.D., JOHNSON, L.P., JONES, T.W., KELLOGG, H.B., RADKE, H.M., SIKKEMA, W.W., JOLLY, P.C. & HAMMAR, S.P. (1985). Participants in prospective randomised clinical trials for resected non-small cell lung cancer have improved survival compared with non participants in such trials. *Cancer*, **56**, 1710-1718.

GILLIS, C.R., HOLE, D.J., STILL, R.M., DAVIS, J. & KAYE, S.B. (1991). Medical audit, cancer registration, and survival in ovarian cancer. *Lancet*, **337**, 611-612.

GRIFFITHS, C.T. (1975). Surgical resection of tumour bulk in the primary treatment of ovarian carcinoma. *Natl Cancer Inst. Monogr.*, **42**, 101-104.

HARDING, M.J., PAUL, J., GILLIS, C.R. & KAYE, S.B. (1993). Management of malignant teratoma. Does referral to a specialist unit matter? *Lancet*, **341**, 999-1002.

HOGBERG, T., CARSTENSEN, J. & SIMONSEN, E. (1993). Treatment results and prognostic factors in a population-based study of epithelial ovarian cancer. *Gynaecol. Oncol.*, **48**, 38-49.

HOLE, D.J. & GILLIS, C.R. (1993). Use of cancer registry data to evaluate the treatment of ovarian cancer on a hospital basis. *Hlth Rep.*, **5**, 117-119.

KARJALAINEN, S. (1990). Geographical variation in cancer patient survival in Finland: chance, confounding, or effect of treatment? *J. Epidemiol. Community Health*, **44**, 210-214.

KARJALAINEN, S. & PALVA, I. (1989). Do treatment protocols improve end results? A study of survival of patients with multiple myeloma in Finland. *Br. Med. J.*, **299**, 1069-1072.

LENNOX, E.L., DRAPER, G.J. & SANDERS, B.M. (1975). Retinoblastoma: a study of natural history and prognosis of 268 cases. *Br. Med. J.*, **3**, 731-734.

MALKASIAN, G.D., DECKER, D.G. & WEBB, M.J. (1975). Histology of epithelial tumours of the ovary: clinical usefulness and prognostic signficance of histologic classification and grading. *Semin. Oncol.*, **2**, 191-201.

MANAGEMENT OF OVARIAN CANCER (1991). Current Clinical Practices, Report of a Working Group. Chairman: Professor J.S. Scott. Standing Subcommittee on Cancer of the Standing Medical Advisory Committee. Department of Health.

OMURA, G.A., BRADY, M.F., HOMESLEY, H.D., YORDAN, E., MAJOR, F.J., BUCHSBAUM, H.J. & PARK, R.C. (1991). Long-term follow-up and prognostic factor analysis in advanced ovarian carcinoma. The Gynaecologic Oncology Group experience. *J. Clin. Oncol.*, **9**, 1138-1150.

RIES, L.A.G., HANKEY, B.F., MILLER, B.A., HARTMAN, A.M. & EDWARDS, B.K. (1991). Cancer Statistics Review 1978-88. National Cancer Institute. NIH Publication No. 91-2789. NCI: Bethseda, MD.

STILLER, C.A. (1988). Centralisation of treatment and survival rates for cancer. *Arch. Dis. Child.*, **63**, 23-30.

STILLER, C.A. & DRAPER, G.J. (1989). Treatment centre size, entry to trials, and survival in acute lymphoblastic leukaemia. *Arch. Dis. Child.*, **64**, 657-661.

VOEST, E.E., VAN HOUWELINGEN, J.C. & NEIJT, J.P. (1989). A meta-analysis of prognostic factors in advanced ovarian cancer with median survival and overall survival measured with log (relative risk) as main objectives. *Eur. J. Cancer Clin. Oncol.*, **27**, 711-720.

WHO (1976). International Classification of Diseases for Oncology. WHO: Geneva.